# Spatiotemporal expansion of *Aedes aegypti* and the dengue fever epidemic under climate change in China

**DOI:** 10.1371/journal.pntd.0013702

**Published:** 2025-11-19

**Authors:** Ziyue Zhou, Guanhao He, Jianxiong Hu, Guodong Li, Hepeng Pan, Yayi Li, Siwen Yu, Zhiqing Chen, Wenjun Ma, Guanghu Zhu, Tao Liu

**Affiliations:** 1 Department of Public Health and Preventive Medicine, School of Medicine, Jinan University, Guangzhou, China; 2 School of Mathematics and Computing Science, Guangxi Colleges and Universities Key Laboratory of Data Analysis and Computation, Guilin University of Electronic Technology, Guilin, China; 3 China Greater Bay Area Research Center of Environmental Health, School of Medicine, Jinan University, Guangzhou, China; 4 Center for Applied Mathematics of Guangxi (GUET), Guilin, China; 5 Key Laboratory of Viral Pathogenesis & Infection Prevention and Control, Jinan University, Ministry of Education, Guangzhou, China; University of Virginia School of Medicine, UNITED STATES OF AMERICA

## Abstract

**Background:**

*Aedes aegypti* is the primary vector of dengue fever, a disease whose transmission is strongly dependent on warm, humid climates. While the geographical and seasonal patterns of dengue are well-established, systematic projections of how climate change will affect the distribution of *Aedes aegypti* and dengue transmission risk in China remain limited.

**Method:**

We integrated a phenological model with a dynamical mathematical model to project the future distribution and population dynamics of *Aedes aegypti* under multiple Shared Socioeconomic Pathways (SSPs). We assessed life-cycle completion (LCC) intensity at the municipal level across China and simulated detailed mosquito population dynamics and dengue transmission in six representative cities under sustainable (SSP1-2.6), regional rivalry (SSP3-7.0), and fossil-fueled development (SSP5-8.5) scenarios.

**Results:**

Climate warming is projected to accelerate the developmental rate of *Aedes aegypti*, with the most severe risks under the SSP5-8.5. By the 2090s, southern coastal cities could average 26 LCCs per year, and approximately 90% of Chinese cities may sustain at least one annual generation. The mosquito’s range is expected to expand northward, with peak abundance shifting to September-October. Lengthened active seasons, particularly in the third and fourth quarters, are anticipated. Consequently, dengue incidence is projected to rise, peaking later in the year (October-November). In a high-emission future, Guangzhou could experience peaks of up to 11,000 daily cases.

**Conclusion:**

Climate warming could increase the generational turnover, expand the geographic range, and prolong the seasonal activity of *Aedes aegypti* in China, thereby significantly elevating the risk of dengue transmission. These projections enhance our ability to predict outbreaks and are critical for informing proactive, targeted public health control strategies.

## Author summary

Dengue fever, a climate-sensitive vector-borne disease, is expanding its global reach. This study projects how climate change will drive the spread of *Aedes aegypti* across China by integrating climate scenarios with phenological and dynamical mosquito models. Our analysis indicates that by the 2090s, projected warming will permit *Aedes aegypti* to complete more life cycles annually, particularly in southern coastal cities where conditions may support year-round breeding. The mosquito’s suitable range is projected to expand northward, with seasonal abundance peaks shifting to late autumn. We project that high-emission scenarios could lead to thousands of daily dengue cases during peak season in major coastal cities like Guangzhou by the century’s end. These shifts suggest that dengue outbreaks may intensify in frequency and severity, underscoring the need for earlier and more geographically targeted control. Our findings provide critical evidence for policymakers and public health officials to develop proactive surveillance and early warning systems, ultimately helping to mitigate the growing dengue burden in a warming China.

## 1 Introduction

*Aedes aegypti* has a strong affinity for preferentially selecting human hosts for blood meals [1]. This mosquito species primarily transmits several internationally concerning vector-borne diseases, including the dengue virus (DENV), Zika virus, yellow fever virus, and chikungunya virus. *Aedes aegypti* is primarily found in Southeast Asia, Africa, South America, and parts of Southern Europe. Meteorological factors such as temperature, precipitation, and humidity play crucial roles in its breeding and spreading. In warmer climates, the life cycle of *Aedes aegypti* is shortened [2], and the transition from immature stages to adult mosquitoes accelerates, indirectly promoting disease transmission. With the effects of global climate change, urbanization, and population growth, an increasing number of urbanized areas are becoming suitable for *Aedes aegypti* breeding. Takuya Iwamura’s phenology model [3] indicated that the area suitable for *Aedes aegypti* growth worldwide increased by approximately 1.5% every ten years during 1950–2000, and invasion fronts in North America and China are projected to accelerate from 2 to 6 km/yr by 2050. Jing’s [4] analysis using Coupled Model Intercomparison Project Phase 5 meteorological data revealed that the potential abundance of *Aedes aegypti* has increased by 9.5% globally over the past century, and the abundance is expected to further increase by 20% and 30% by the end of this century under low-carbon and high-carbon emission scenarios, respectively.

DENV is one of the most widely spread mosquito-borne arboviruses in the world, belonging to the genus Orthoflavivirus in the family Flaviviridae [5], and DENV is mainly transmitted by *Aedes aegypti* and *Aedes albopictus*. Dengue fever is a disease caused by infection with DENV, infecting at least 50–100 million people globally each year, of whom at least 500,000 require hospitalization, and there are significantly more patients with latent infections than overt ones [6]. Abundant studies indicated that temperature was positively correlated with dengue cases [7]. The suitable temperature for the transmission of DENV is from 26°C to 33°C [8–10], while the optimal temperature range for the growth and development of *Aedes aegypti* is between 25°C and 30°C [11,12]. *Aedes aegypti* exhibits greater thermal adaptability than *Aedes albopictus*, maintaining DENV transmission capability across a wider temperature range compatible with projected global warming [11]. This suggests that with rising temperatures in the future, *Aedes aegypti* may have a competitive advantage [13]. Furthermore, *Aedes aegypti* is more efficient at transmitting DENV than *Aedes albopictus* [14], and a 10°C increase in temperature leads to a halving of the bite interval, increasing transmission by at least 2.4-fold [15].

In China, the provinces reporting dengue cases have expanded from the Southeastern coastal areas to the Southwestern, Central, Northeastern, and Northwestern regions. Among them, Guangdong, Guangxi, Yunnan, Fujian, and Hainan have higher incidence rates [16]. Under the current climate conditions, dengue in China is primarily transmitted by *Aedes albopictus*. However, with climate warming, *Aedes aegypti* is also spreading to regions such as Hainan, southern Guangdong, and southern Yunnan. From July to October, its abundance gradually increases across various regions [17]. However, the research on *Aedes aegypti* is limited to observational data from narrow areas, lacking systematic studies on its future distribution and impact on dengue in the context of climate change. This study therefore focuses on *Aedes aegypti* to address these gaps and lay the groundwork for effective control strategies by investigating: (1) in-depth exploration of the impact of future climate change on the distribution of *Aedes aegypti* in China; and (2) exploration of climate change-driven spatial and temporal variations in the burden of disease caused by the proliferation of *Aedes aegypti* in dengue fever.

China’s complex and diverse climate, combined with the wide thermal tolerance of *Aedes aegypti*, is expected to enhance the mosquito’s competitiveness under future warming [18]. We therefore hypothesize that climate change will significantly increase the number of annual life-cycle completions (LCC) of *Aedes aegypti* at the municipal level, drive a northward and westward expansion of its range, prolong its seasonal activity, and lead to a higher dengue fever burden, with these effects being most pronounced under high-emission scenarios.

To this end, this study uses phenological and mathematical models, combined with meteorological data, to study the distribution and spread of *Aedes aegypti* in China. We first analyze the spatial and temporal changes of *Aedes aegypti* at the municipality level in China, then refines the aquatic and adult stages of the lifecycle, introducing puddle dynamics to model population competition. The study also uses a human-mosquito coupled model to simulate the transmission of DENV in six cities of China. This research fills the gap in small-scale distribution data of *Aedes aegypti* in China, providing new insights for mosquito vector control and dengue fever prediction. The model can also be extended to study the transmission dynamics of other mosquito-borne diseases.

## 2 Method and material

### 2.1 Study settings

In this modeling study, the unit of data analysis was defined as a municipality, prefecture, or county directly under a province (hereinafter referred to as a city). The Qinling-Huaihe Line was used to divide China into Southern and Northern regions. The phenological model was developed using municipality level data to establish distribution patterns. For the dynamic transmission model, six representative cities were selected as case studies (Fig A in S2 Appendix). The selection was based on three primary criteria aligned with the study’s objectives: (1) to capture a clear latitudinal and climatic gradient across Southern China, which is critical for assessing the northward expansion of *Aedes aegypti*; (2) to include cities that are established sentinel sites for *Aedes aegypti* surveillance in China [19], ensuring relevance to national public health monitoring; and (3) to represent regions with a history of mosquito-borne disease studies, providing a contextual background for interpreting model outcomes [20]. Specifically, Guangzhou represents the high-risk Guangdong-Hong Kong-Macao Greater Bay Area, Haikou represents the tropical region, Nanning represents the central and western regions, Guiyang represents the southwestern region, Changsha represents the central China region (a potential northern frontier for expansion), and Fuzhou represents the eastern coastal region. This study, including data processing, model development, and analysis, was conducted between August 2023 and December 2024.

### 2.2 Sources of data

The daily average temperature $T_{m}$, daily maximum temperature $T_{max}$, daily minimum temperature $T_{min}$, precipitation $P_{r}$, and population data at municipality level across China from 2030 to 2100 were obtained from the latest Coupled Model Intercomparison Project Phase 6 (CMIP6) developed by the Inter-Sectoral Impact Model Intercomparison Project (ISI-MIP, https://www.isimip.org/) (Table A in S3 Appendix). The CMIP6 integrated scenarios rooted the Shared Socioeconomic Pathways (SSPs) [21]. In this study, the three SSPs were selected to encompass the full spectrum of potential climate futures: SSP126 (sustainable pathway), SSP370 (regional rivalry pathway), and SSP585 (fossil-fueled development pathway). These scenarios enable assessment of how varying greenhouse gas emission (GGE) levels may differentially impact *Aedes aegypti* distribution and dengue transmission dynamics). SSP126 represents a social development model with low level of GGE. In this scenario, the changes in radiative forcing (RF) are expected to get to 2.6 W/m^2^ by 2100; SSP370 assumes the changes in change in RF are expected to get to 7.0 W/m^2^ by 2100, which indicates a model with middle-high level of GGE; SSP585 is a development model with high level of GGE, and it is considered as the worst development trajectory in the future with an expected change in RF of 8.5 W/m^2^ by 2100 [22]. This gradient enables evaluation of both best- and worst-case scenarios while capturing intermediate outcomes.

In each scenario, we chose the GFDL-ESM4 model from CMIP6, with future climate and population data under the “r1i1p1f1” scenario. The data (Table B in S3 Appendix) was filtered to extract the daily average temperature $T_{m}$, daily maximum temperature $T_{max}$, daily minimum temperature $T_{min}$, precipitation $P_{r}$, population data, and geographical location data for China at the municipality level during the period from 2030 to 2100. The temperature data was then converted from Kelvin to Celsius, and the precipitation data per second was aggregated into daily precipitation. This prepared data was later used for modeling and projection onto maps, making it capable of accurately describing the historical and future temperatures of China [23]. The grid has a spatial resolution of 0.25° × 0.25° and provides daily temporal resolution. This dataset has been extensively used in climate change studies on mosquito vectors, consistently affirming its reliability [24].

### 2.3 Analyzing the impact of climate change on the spatiotemporal distribution of *Aedes aegypti* based on phenological models

A phenological model of *Aedes aegypti* proposed by Takuya Iwamura [3] was applied to estimate the nuanced effects of temperature variations at different stages on the development rates of various life stages of the mosquito (including larvae, pupae, and adults). Subsequently, under three climate change scenarios, we estimated the number of life cycle completions (LCC) of *Aedes aegypti* at municipality level across China from 2030 to 2100, representing the number of generations completed in a given unit of time.

**Fig 1:**
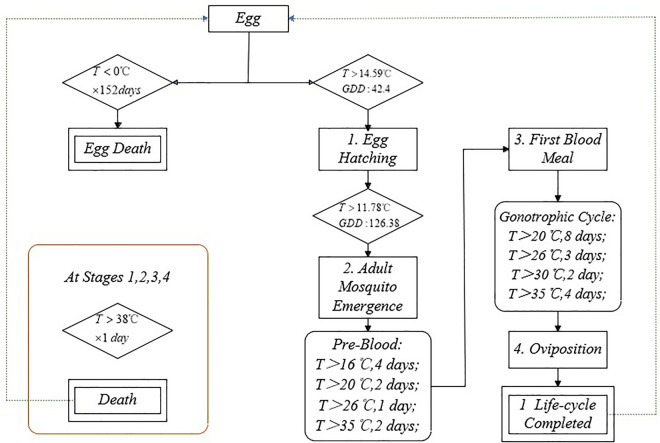
Flowchart of *Aedes aegypti* phenological model. Rectangles represent different developmental stages, while diamonds indicate specific developmental conditions.

The phenological model included the aquatic stage (immature phase) and the aerial stage (adult phase) of *Aedes aegypti*. The aquatic stage consists of four developmental stages: eggs, larvae, pupae, and adult emergence, while the aerial stage includes four adult phases: mating, blood feeding, pregnancy, and oviposition. To simplify the life cycle [25], the phenological model merged and reclassified the aquatic stage and the aerial stage into four sub-stages: egg hatching stage, immature development stage (larvae and pupae), blood feeding stage, and oviposition stage (S1 Appendix). Similar to other studies [26], the model only considers female *Aedes aegypti* as females are the sex that lays eggs and takes blood meals, and it assumes that under suitable environmental conditions, there are always enough male to fertilize the females. The model is based on the temperature thresholds, GDD (Growing Degree Days) and developmental rates required for these four main developmental stages. The thresholds define the upper or lower temperature conditions that must be met for growth and development to continue or cease, and GDD refers to the accumulation of daily average temperatures exceeding the temperature threshold and remaining below the maximum temperature of 35°C [3]; no GDD accumulates above this maximum temperature, with the following calculation formula,


$$GDD=\sum_{i}\left(T_{i}-T_{thr}\right)$$

where $T_{i}$ represents the daily average temperature on the *i*-th day since the start of the life stage (with an upper limit of 35°C), and $T_{thr}$ is the temperature threshold for development. For example, if $T_{i}<T_{thr}$, then $T_{i}-T_{thr}=\text{0}$ to reflect that the accumulation of GDD cannot be negative. When $T_{i}>\text{3}{\text{5}}^{\circ }\text{C}$, then $T_{i}=\text{3}{\text{5}}^{\circ }\text{C}$. If the calculated GDD exceeds the established GDD required to complete that life stage, the model progresses to the next life stage. If not, the model cannot enter the next life stage and will continue to accumulate GDD in the current stage (Fig 1). The phenological model established for *Aedes aegypti* in this section is an idealized growth and development model, assuming that only climatic factors influence the mosquito’s generation, without considering external human interventions or other factors.

### 2.4 Temperature and precipitation-dependent dynamics of *Aedes aegypti* population abundance based on a dynamic model

We used a system of ordinary differential equations (ODE) to assess the impacts of temperature and precipitation on the abundance of *Aedes aegypti* population:


$$\frac{dE_{d}}{dt}=\left(\text{1}-p_{w}\right)\phi A_{\text{2}}-\sigma_{ed}E_{d}-\mu_{ed}E_{d}$$

$$\frac{dE_{w}}{dt}=p_{w}\phi A_{\text{2}}+\sigma_{ed}E_{d}-\sigma_{ew}E_{w}-\mu_{ew}E_{w}$$

$$\frac{dL}{dt}=\sigma_{ew}E_{w}-\mu_{l}\left(\text{1}+\frac{L}{K}\right)L-\sigma_{l}L$$

$$\frac{dP}{dt}=\sigma_{l}L-\mu_{p}P-\sigma_{p}P$$

$$\frac{dA_{\text{1}}}{dt}=\frac{\text{1}}{\text{2}}\sigma_{p}P-\left(d_{a}+n_{a}\right)A_{\text{1}}+\frac{\text{1}}{\text{2}}\frac{\text{1}}{l_{v}}A_{\text{2}}$$

$$\frac{dA_{\text{2}}}{dt}=n_{a}A_{\text{1}}-d_{a}A_{\text{2}}-\frac{\text{1}}{l_{v}}A_{\text{2}}$$

$$\frac{dA_{\text{3}}}{dt}=\frac{\text{1}}{\text{2}}\frac{\text{1}}{l_{v}}A_{\text{2}}-d_{a}A_{\text{3}}$$

This describes the population dynamics of *Aedes aegypti* across seven stages of its lifecycle: dry eggs, wet eggs, larvae, pupae, and three stages of adult mosquitoes (Fig B in S2 Appendix). Previous studies have illustrated that temperature significantly affects the duration of the egg-laying period $\left(l_{v}\right)$, the maturation and mortality rates of larvae and pupae $\left(\sigma_{l},\;\sigma_{p},\;\mu_{l},\;\mu_{p}\right)$, and the daily mortality rate of adult female $\left(d_{a}\right)$ [27], all of which are listed in expression form, and other parameters are listed as constant values, while rainfall plays an important role in providing breeding sites such as the environmental carrying capacity (Κ). Therefore, the impacts of these factors are incorporated into the model (Tables C and D in S3 Appendix). Critically, a key innovation of our modeling approach is the explicit integration of puddle dynamics to better capture how rainfall patterns influence breeding site availability, a factor often simplified in previous models. This allows us to more realistically simulate the population dynamics of *Aedes aegypti* across its seven life stages under varying climatic conditions.

Therefore, puddle dynamics are introduced into a system of ODE to describe the population dynamics of the seven stages. According to previous references [28,29], the environmental carrying capacity (K) for aquatic larvae is a function of the moisture in the breeding sites, denoted by the amount of water in the puddle $V_{pond}$, such that $\text{K}=L_{max}\times V_{pond}$, where $L_{max}$ is the maximum larval biomass per unit surface area of water. In subsequent studies, $L_{max}$ is set to 300 mg m^-2^ [29,30]. Research has shown [30,31] that heavy rainfall can wash away breeding sites, resulting in high mortality rates for larvae. Even outside the rainy season, it can be assumed that puddles exist in irrigated areas. Therefore, this study considers the dynamics of puddles:


$$\frac{dV_{pond}}{dt}=\kappa_{v}\left[R_{f}\left(V_{max}-V_{pond}\right)-V_{pond}\left(\vartheta +I_{f}\right)\right]$$

Where $V_{pond}$ ranges between the minimum and maximum water volumes, $V_{min}\leq V_{pond}\leq V_{max}$. The daily rainfall/precipitation rate is denoted by $R_{f}$, $\kappa_{v}$ represents the geometric shape of the puddle, ϑ indicates the daily evaporation rate, and $I_{f}$ is the daily infiltration rate. According to reference [28], a fixed constant parameter is assigned to the infiltration rate. It follows from the Hamon formula [32] that


$$\vartheta =\text{2}.\text{1}\times H_{t}^{\text{2}}\times \left(\frac{e_{s}}{T+\text{2}\text{7}\text{3}.\text{3}}\right)$$

where $H_{t}$ represents the number of daylight hours per day. In a study focused on the South African region [26], the range of daylight hours is between 10–14 hours per day; for simplification in this analysis, it is set as a constant of 10.5 hours per day. $e_{s}$ denotes the saturation vapor pressure with


$$e_{s}(T)=\text{0}.\text{6}\text{1}\text{0}\text{8}e^{\left(\frac{\text{1}\text{7}.\text{2}\text{7}T}{T+\text{2}\text{3}\text{7}.\text{3}}\right)}$$

### 2.5 Exploring the impact of *Aedes aegypti* abundance on dengue transmission based on a human-mosquito coupling model

This section proposes a human-mosquito coupling model to explore the impact of *Aedes aegypti* abundance on dengue transmission. In this model, we introduce *Aedes aegypti* abundance obtained in the previous section, considering the provincial capital cities as homogeneous epidemiological regions, with the population of each region also incorporated into the model.


$$\frac{dO_{I}}{dt}=\lambda_{M}\tau A_{\text{1}}-d_{a}O_{I}$$

$$\frac{dS}{dt}=-\lambda_{\text{1}}S$$

$$\frac{dE}{dt}=\lambda_{\text{1}}S-\delta_{h}E$$

$$\frac{dI}{dt}=\delta_{h}E-\gamma_{h}I$$

$$\frac{dR}{dt}=\gamma_{h}I$$

In this model, the provincial capital cities are considered as a block, and individuals are classified based on their health status into susceptible (S), exposed (E), infected (I), and recovered (R) categories, and the susceptible *Aedes aegypti* $\left(A_{\text{1}}\right)$ become infected (entering the $O_{I}$ compartment) by biting infectious humans, with the infection rate depending on the transmission potential ($\lambda_{M})$ and the duration of the extrinsic incubation period (τ).

For humans, this section defines four stages, incorporating the total population of provincial capital cities into the mathematical model. A susceptible individual can become infected with the DENV through a bite from an infectious *Aedes aegypti*. The susceptible population (S) acquires primary infection at a rate of $\lambda_{\text{1}}$ (Fig C in S2 Appendix). The variables and parameters included in the system equations, along with their units and derivation sources are listed in Tables E and F in S3 Appendix. The temperature-related parameters, $c_{v}$, $\beta_{\text{1}}$ and $\beta_{M}$ are presented in expression form, while all other parameters, τ, $\sigma_{M}$, $\sigma_{\text{1}}$, the intrinsic incubation period after human infection $\left(\delta_{h}\right)$, and the recovery rate after human infection $\left(\gamma_{h}\right)$ are listed as constant values.

## 3 Results

### 3.1 Spatiotemporal changes of *Aedes aegypti* LCC in the future across China

**Fig 2:**
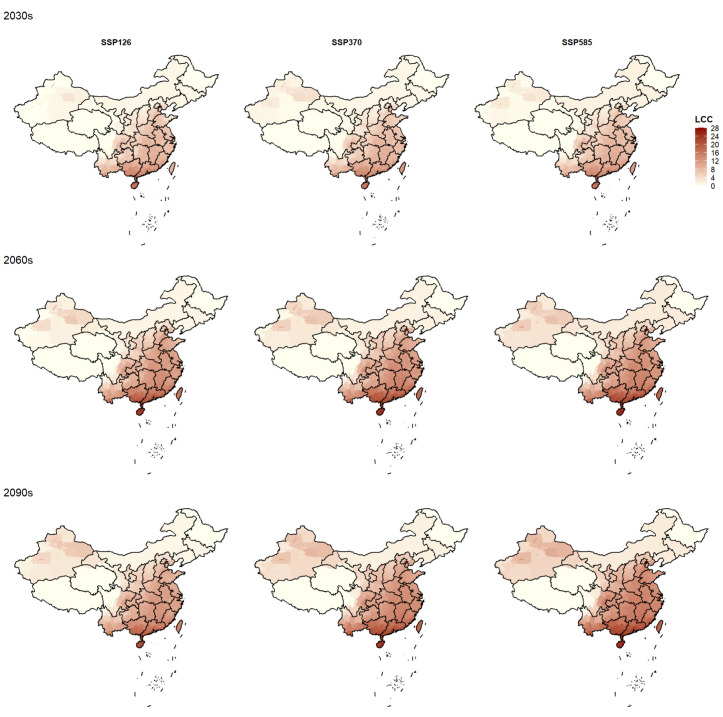
Trend of *Aedes aegypti* LCC values in various cities in China under the SSP126, SSP370, and SSP585 for the 2030s, 2060s, and 2090s. Note: The base map of China (including the national boundary and coastline) was obtained from the Standard Map Service of the Ministry of Natural Resources of the People’s Republic of China (http://bzdt.ch.mnr.gov.cn/) and is authorized for public use and publication.

Fig 2 shows the spatiotemporal changes of *Aedes aegypti* LCC in the future across China. Temporally, it is projected a significant increase in the *Aedes aegypti* LCC from 2030s to 2090s across China, particularly under the SSP370 and SSP585 scenarios. For example, the national average LCC would increase by 4 generations from the 2030s to the 2090s under the SSP585 scenario in China, whereas the national average LCC would increase by 3 generations under the SSP126 scenario. Spatially, it is estimated greater increase in the *Aedes aegypti* LCC in the Southern regions (referring to areas south of the Qinling-Huaihe Line) than in other regions. For example, the *Aedes aegypti* LCC would increase by 26 generations in Guangdong and Hainan from 2030s to 2090s under the SSP585 scenario. By contrast, the *Aedes aegypti* LCC would increase by 3 generations in Beijing and Shandong.

**Fig 3:**
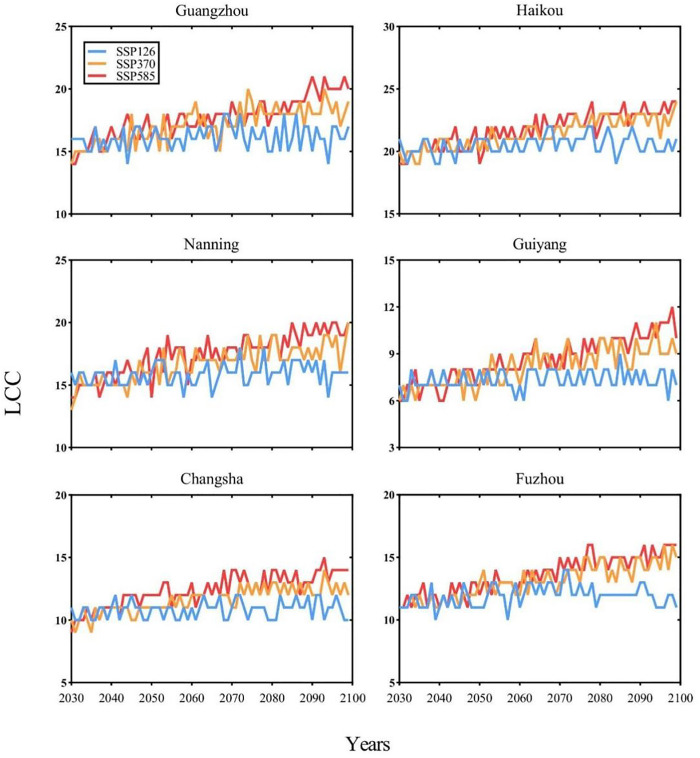
Changes in *Aedes aegypti* LCC in six cities under different SSPs from 2030 to 2100. Y-axis scales vary across subplots to optimize visualization of city-specific trends.

Fig 3 projects the temporal changes of yearly *Aedes aegypti* LCC during 2030–2100 in six capital cities located around 22°N latitude. The model estimates a greater increase in *Aedes aegypti* LCC under the SSP585 and SSP370 scenarios than under the SSP126 scenario. The projected decadal increase rates of LCC during 2030–2100 are as follows: in Guangzhou, 9.2%, 53.1%, and 71.3% under SSP126, SSP370, and SSP585, respectively; in Haikou, 9.4%, 49.2%, and 59.3%; in Nanning, 9.7%, 55.6%, and 71.9%; in Guiyang, 6.0%, 45.7%, and 62.6%; in Changsha, 6.1%, 40.6%, and 66.9%; and in Fuzhou, 10.0%, 57.9%, and 66.8% under the same scenarios.

### 3.2 Temporal changes of *Aedes aegypti* abundance in the future

**Fig 4:**
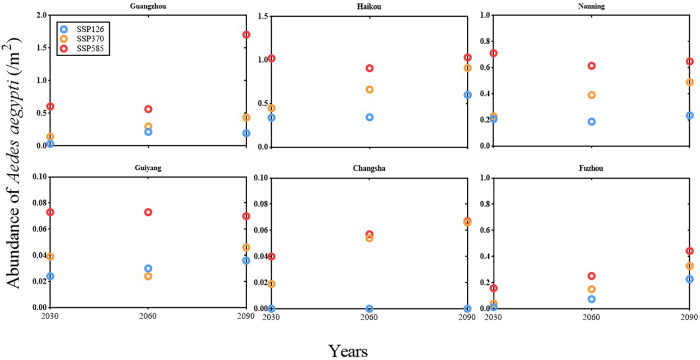
Modeled-projected changes in the average daily number of *Aedes aegypti* abundance from July to December per square meter in six cities under SSP126, SSP370, and SSP585 for the years 2030, 2060, and 2090. Note: Y-axis scales vary across subplots to optimize visualization of city-specific trends.

Fig 4 projects the temporal changes of *Aedes aegypti* abundance (individuals/m²) from July to December under future scenarios across six capital cities. Greater increases in abundance are projected under SSP370 and SSP585 compared to SSP126, with more pronounced rises observed in southeastern coastal cities. For example, in Guangzhou, average daily abundance is projected to increase from 0.61 in 2030 to 1.70 in 2090 under SSP585, and from 0.03 to 0.20 under SSP126. In Haikou, abundance rises from 1.02 in 2030 to 1.03 in 2090 under SSP585, and from 0.34 to 0.60 under SSP126. In Nanning, values shift from 0.71 in 2030 to 0.65 in 2090 under SSP585, and from 0.21 to 0.24 under SSP126. In Guiyang, abundance remains stable at 0.07 under SSP585 between 2030 and 2090, while under SSP126 it increases slightly from 0.02 to 0.04. In Changsha, under SSP585, abundance increases from 0.04 in 2030 to 0.07 in 2090, but remains at 0 throughout under SSP126. In Fuzhou, under SSP585, abundance rises from 0.16 in 2030 to 0.44 in 2090, and under SSP126 it increases from 0.01 to 0.23. These projections highlight notable inter-city variations, with coastal cities such as Guangzhou and Haikou experiencing higher increases compared to more inland or northern cities like Guiyang and Changsha, illustrating a clear gradient of impact from southeastern coastal areas toward central and southwestern regions.

**Fig 5:**
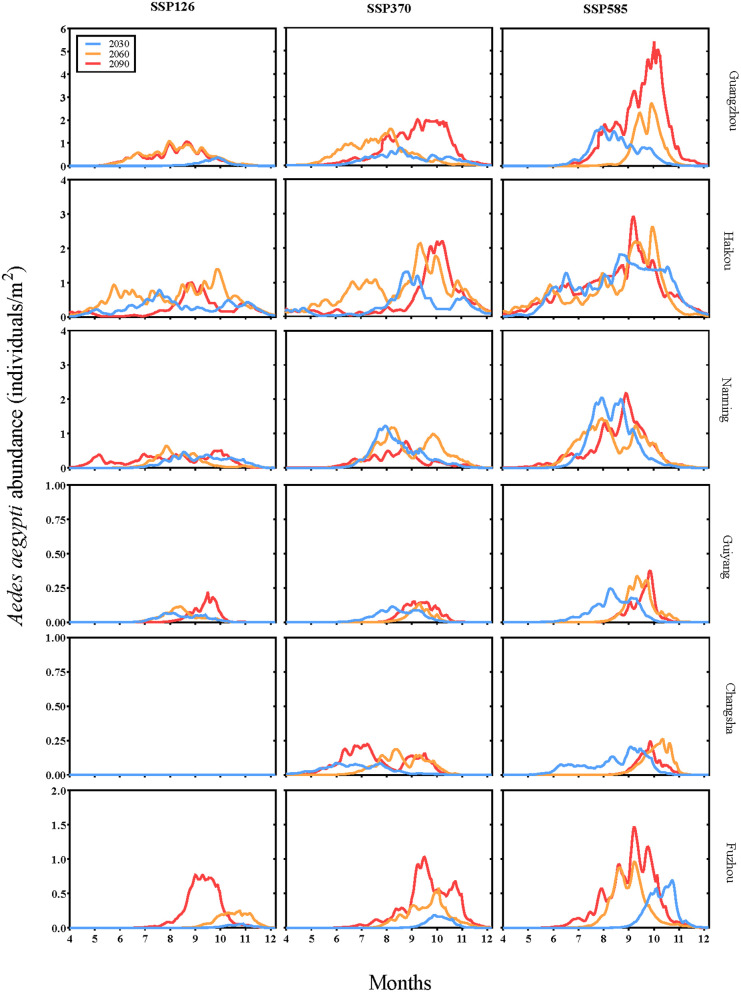
Simulated variation curves of the daily *Aedes aegypti* abundance per square meter in six cities under SSP126, SSP370, and SSP585 for the years 2030, 2060, and 2090. Note: Y-axis scales vary across subplots to optimize visualization of city-specific trends.

Fig 5 projects the seasonal changes of *Aedes aegypti* abundance (individuals/m²) under future scenarios in six capital cities. The simulations indicate that mosquito abundance is generally highest between August and October. Under SSP585 in 2090, abundance in Guangzhou is estimated to rise from August and peak in early October at approximately 5 individuals/m². Similarly, Haikou reaches its peak abundance around October at 0.25 individuals/m², while Nanning peaks near September at about 2 individuals/m². Guiyang also peaks around September with approximately 0.3 individuals/m², Changsha around October at 0.25 individuals/m², and Fuzhou near September at about 1.5 individuals/m². The model outputs further suggest a progressive extension of the seasonal activity window, increasing from 3 months in 2030–4 months by 2060, and reaching 5 months by 2090.

### 3.3 Prediction of dengue fever cases

**Fig 6:**
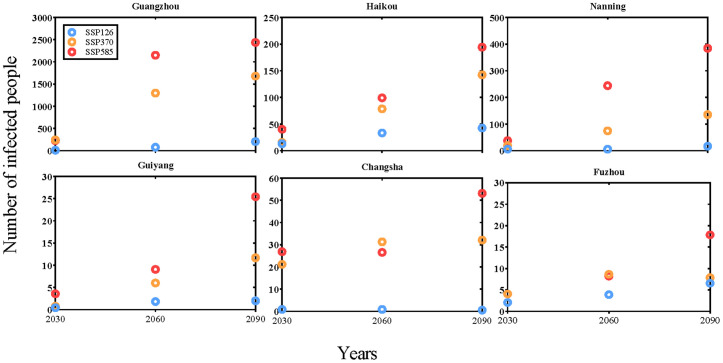
Model-projected changes in the average daily dengue cases from July to December for the years 2030, 2060, and 2090 under the SSP126, SSP370, and SSP585 across six cities. Note: Y-axis scales vary across subplots to optimize visualization of city-specific trends.

Fig 6 presents the model-projected average daily dengue cases attributed to *Aedes aegypti* transmission from July to December under different scenarios across six cities. Under the SSP585 scenario, the simulated average number of dengue cases in Guangzhou is projected to increase from 229 in 2030–2,438 in 2090, while under SSP126, the increase is more moderate, rising from approximately 12–206. In Haikou, cases are projected to rise from 40 to 194 under SSP585 and from 13 to 42 under SSP126. In Nanning, the projected increase under SSP585 is from 37 to 384, compared to an increase from 6 to 16 under SSP126. In Guiyang, case numbers are projected to increase slightly from 3 to 5 under SSP585 and from 0 to 1 under SSP126. In Changsha, under SSP585, the average daily cases are projected to rise from 26 in 2030–53 in 2090, while under SSP126 they are expected to remain near zero. In Fuzhou, the model projects an increase from 4 to 17 under SSP585 and from 2 to 6 under SSP126.

**Fig 7:**
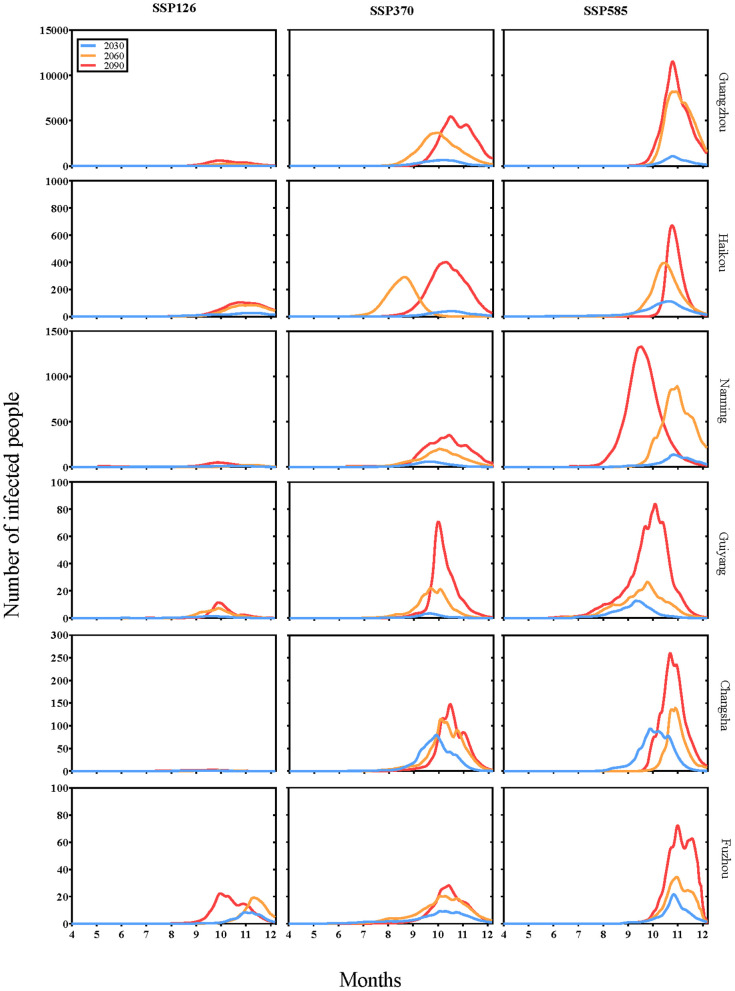
Model-projected changes in the daily dengue cases for six cities under the SSP126, SSP370, and SSP585 for the years 2030, 2060, and 2090. Note: Y-axis scales vary across subplots to optimize visualization of city-specific trends.

Fig 7 illustrates the simulated seasonal trends in the daily number of incident dengue cases in six cities. The model projections suggest the peak number of daily cases occurs between September and November. For example, in Guangzhou in 2090 under the SSP585 scenario, the estimated daily case count is projected to begin rising in September and reach a peak of approximately 11,000 new cases per day between October and November. Similarly, Haikou is projected to peak during October-November with around 700 new daily cases, while Nanning is expected to reach its peak around September-October with approximately 1,400 new cases per day. Guiyang is projected to peak during October-November with about 80 new daily cases, Changsha around October-November with approximately 270 new cases per day, and Fuzhou during October-November with around 70 new cases per day. This peak in infections is estimated to lag approximately one month behind the peak in *Aedes aegypti* abundance. With future climate warming, the epidemic season suggests an expanding trend, increasing from 2 months under SSP126, to 3 months under SSP370, and further to 4 months under SSP585. Additionally, over time, the simulated duration of the epidemic in 2090 has extended by an average of 2 months compared to 2030 across all scenarios, and the peak daily case incidence has roughly doubled.

### 3.4 Sensitivity analysis

Since the model parameters are related to temperature, using Guangzhou in 2060 under the SSP370 as an example, both temperature increase and decrease will affect the abundance of *Aedes aegypti* and the transmission of dengue fever (Fig D in S2 Appendix). Compared to the simulated real temperature, 1°C increase in temperature will lead to a 6.10% increase in the average abundance of *Aedes aegypti* (individuals/m²) and a 66.70% increase in the average number of dengue fever cases during July to December. A 1°C decrease in temperature will result in a 33.92% decrease in the average abundance of *Aedes aegypti* and a 40.39% decrease in the average number of dengue fever cases. When the temperature increases by 2°C, *Aedes aegypti* will become less active due to excessively high temperatures, with a 52.47% decrease in average abundance and a 39.31% decrease in average dengue fever cases. A 2°C decrease in temperature will lead to a 40.60% decrease in the average abundance of *Aedes aegypti* and a 51.34% decrease in the average number of dengue fever cases.

## 4 Discussion

This study utilized a phenological model of *Aedes aegypti*, combined with a refined mathematical model and a human-mosquito coupling model, to assess the impacts of different climate change scenarios on the spatiotemporal expansion of *Aedes aegypti* and the dengue fever in China. We estimated that the future global warming particularly under the SSP370 and SSP585 would significantly increase the survival and abundance of *Aedes aegypti*, the potential distribution of *Aedes aegypti* is projected to extend substantially into Northern and Western regions, and the risks of DENV transmitted by *Aedes aegypti* would be greater from August and November. These findings strongly indicate the spatiotemporal expansion of DENV transmitted by *Aedes aegypti* in future China, which provides significant information of making targeted measures to control *Aedes aegypti* and dengue in the future.

We first projected that the global warming would significantly increase the survival and abundance of *Aedes aegypti*, which could subsequently elevate the risk of dengue fever epidemics in China. Several previous studies also reported significant effects of global warming on the *Aedes aegypti* and dengue fever. For example, Jing [4] estimated that the increase in the abundance of *Aedes aegypti* is closely related to the rise in global temperatures. From the last century to the present, the estimated global potential abundance change is an increase of 9.5%. In comparison, future scenarios with low and high carbon emissions will further increase the abundance of *Aedes aegypti* by 20% or 30%, this is because uncontrolled greenhouse gas emissions shorten the lifecycle, providing favorable climatic conditions for its reproduction and expansion. Wu [33] used spatial analysis and found that the spread of dengue fever is closely related to climate change. The results indicated that for every 1°C increase in the monthly average temperature, the total population at risk of dengue fever transmission would increase by 1.95 times. In summary, climate change promotes the growth, reproduction, and activity of *Aedes aegypti*, while also maintaining the DENV activity within a favorable range, thereby significantly increasing the risk of dengue transmission. However, it is noteworthy that extreme temperature increases beyond the optimal range can have an inverse effect [20], which evidences reduced mosquito fitness and viral replication under such extreme thermal conditions.

We further estimated that the *Aedes aegypti* and its transmitted dengue fever would expand to Northern and Western China under all climate change scenarios. Several previous studies also found spatial expansion of *Aedes aegypti* and dengue fever worldwide. By 2050, the abundance of *Aedes aegypti* will increase in temperate regions, tropical regions, and even in high-altitude areas previously protected by low temperatures [34]. By 2080, the number of cases transmitted by *Aedes aegypti* is expected to be nearly 1 billion higher than it is today [34]. These findings suggest that climate change will have a profound impact on the global distribution and burden of infectious diseases.

Furthermore, our results indicated that the seasonal presence of *Aedes aegypti* shows an expanding trend, increasing from 3 months in 2030, to 4 months in 2060, and further to 5 months by 2090. Dengue epidemics transmitted by *Aedes aegypti* primarily occur between September and November, and with future climate warming, the epidemic season expands from 2 months under SSP126, to 3 months under SSP370, and to 4 months under SSP585. Over time, the duration of the epidemic in 2090 has extended by an average of 2 months compared to 2030 across all scenarios, and the peak number of infections has doubled. This is consistent with previous studies [35]: dengue fever occurs in Southwestern regions, Southeastern coastal areas, and Inland regions. These outbreaks typically occur between June and November, with peaks usually in September or November. This peak occurs approximately 1 month later than the peak abundance of mosquito vector.

Climate change leads to changes in temperature and precipitation patterns, which directly affect mosquito habitats and alter key parameters such as growth rate, reproduction cycles, and survival rates. However, climate models have inherent uncertainties, particularly in terms of the accuracy of predicting future climate changes across different regions, and parameters can vary across regions, making these differences difficult to predict precisely. Furthermore, different climate change scenarios will produce varying results. Choosing the appropriate scenario for modeling is an important source of uncertainty. The model used in this study integrates several significant influencing factors, such as climate conditions and population factors, to predict the future expansion trends of *Aedes aegypti* in China and the potential dengue fever burden it may cause. This provides new insights for mosquito control and enhances the predictability and risk assessment of dengue fever outbreaks.

The exploratory projections obtained from the models in this study could contribute to enhancing our understanding of the lifecycle of *Aedes aegypti* and may offer preliminary evidence for informing mosquito control efforts. In the long term, reducing emissions and mitigating the effects of climate change are critical to slowing the ongoing trends that exacerbate the spread of vector-borne diseases [36]. However, in the short term, the reality is that we cannot reverse the ongoing trends of climate change immediately. Therefore, public health systems must be proactive in adapting to these changes. Early and targeted interventions are crucial to mitigate the impact of climate-induced shifts in mosquito populations [37]. This includes improving surveillance systems to monitor *Aedes aegypti* populations more effectively, using predictive models to identify high-risk areas, and implementing focused mosquito control measures [38].

This study provides several key advancements that distinguish it from previous work and address critical research gaps in fine-scale projections of *Aedes aegypti* distribution under climate change. First, it employs a municipality-level analysis across China, achieving an unprecedented spatial resolution that moves beyond regional or provincial scales common in existing literature. This granular approach allows for more targeted and actionable public health planning. Second, unlike studies that focus solely on vector ecology or disease transmission, this work integrates phenological models with a dynamical transmission framework to simultaneously project the impact of climate change on both *Aedes aegypti* abundance and dengue fever incidence, providing a more holistic view of future risk. Third, a significant novel aspect is the mechanistic incorporation of puddle dynamics into the population model. By refining the aquatic and adult stages and explicitly modeling breeding site availability and persistence in response to rainfall, this study offers a more biologically realistic representation of the lifecycle, thereby substantially improving the accuracy of abundance projections. Collectively, these advancements fill a critical gap in forecasting the spatiotemporal distribution of *Aedes aegypti* in China under future climate scenarios, laying an essential theoretical foundation for developing targeted and effective prevention and control measures.

While this study focuses on climate-driven projections, predicted results have important implications for public health preparedness. The projected northward expansion of *Aedes aegypti* and lengthening transmission seasons under high-emission scenarios suggest that current intervention strategies may need to be adapted and expanded geographically and temporally [16]. For instance, our models indicate that regions such as Changsha and Guiyang may become newly vulnerable, necessitating enhanced surveillance and preemptive vector control measures. Similarly, the extended transmission season suggests that control programs may need to operate over longer periods. Future work could explicitly integrate intervention scenarios (e.g., the effect of larval source management) into the modeling framework to quantify the potential for mitigating climate-induced risks [39]. Our projections thus provide a baseline against which the effectiveness of such public health interventions can be evaluated.

Several limitations exist in this study that need to be clarified. (1) Our projections of *Aedes aegypti* LCC and abundance are based on the assumption of no human interventions (e.g., vector control programs) and do not account for species competition. Incorporating inter-species dynamics, particularly in regions with diverse vector populations like Brazil, could improve the accuracy of future abundance estimates [40]. (2) The biological parameters used in the model were sourced from the literature, where previous researchers fitted the corresponding parameters using observational data from specific regions, which may make the results more aligned with local conditions. However, currently, there are no specific expressions for parameters such as the transmission efficiency of *Aedes aegypti* in relation to dengue fever in China. (3) Our model did not account for all potential influencing factors, such as socioeconomic conditions, future population movements, vaccine usage, or stochastic events. The inclusion of factors like GDP, as demonstrated in other studies [4], could be considered in future work.

The model represents a proof-of-concept framework that awaits future validation with empirical data as surveillance for *Aedes aegypti* expands in China. Furthermore, a comprehensive sensitivity analysis encompassing a wider range of parameters and model assumptions will be conducted as part of this validation process. Future research could consider these areas: First, empirical studies on *Aedes aegypti* life-cycle parameters under Chinese climatic conditions are urgently needed to localize developmental thresholds (e.g., temperature-dependent egg hatching rates) and validate model assumptions. Second, incorporating artificial intelligence and machine learning methods into vector monitoring or disease burden predictions to mutually validate the results of dynamic models, thereby improving their predictive capability and utility in addressing the impacts of climate change. Additionally, regional cooperation centers could be established to predict future disease burdens under different climate change scenarios in various regions, assisting low- and middle-income countries in preparing for the increasing challenges of infectious diseases posed by climate change [41]. The model proposed in this study could also be expanded to investigate the dynamics of other mosquito populations and the transmission of other mosquito-borne diseases.

## 5 Conclusions

Our study suggests that global climate warming is projected to accelerate the growth and development rate of *Aedes aegypti* in China, with the highest risk projected under the SSP585 scenario. Spatially, climate warming is expected to expand the distribution range of mosquitoes, pushing them northward and westward. Seasonally, the activity season of mosquitoes will be prolonged, with the most significant impacts in the third and fourth quarters. The projected increase in *Aedes aegypti* abundance could lead to a rise in dengue fever incidence, with peak infection periods occurring from October to November. Furthermore, future climate warming may extend the epidemic season. These model-derived insights provide new insights into mosquito control and enhance the predictability and risk assessment capabilities for dengue outbreaks. The results suggest that early and targeted interventions could be crucial for mitigating the impact of climate change on mosquito population changes. This includes improving monitoring systems, more effectively tracking *Aedes aegypti* populations, using predictive models to identify high-risk areas, and implementing targeted mosquito control measures.

## Supporting information

S1 Appendix Description of mosquito growth and dengue transmission. (DOCX)

S2 Appendix **Fig A.** Geographical distribution of the six case-study cities across China. **Fig B.** Flowchart of *Aedes aegypti* population model. **Fig C.** Dynamics flowchart of dengue virus transmission at the human - *Aedes aegypti* Interface. **Fig D.** Sensitivity of *Aedes aegypti* abundance and dengue fever cases to temperature variations under the SSP370 Scenario (Guangzhou, 2060).
(DOCX)

S3 Appendix **Table A.** Summary of shared socioeconomic pathways. **Table B.** Variable description in NEX-GDDP-CMIP6. **Table C.** Description of variables related to *Aedes aegypti.* **Table D.** Description of parameters related to *Aedes aegypti.* **Table E.** Description of variables related to the human-mosquito coupling model. **Table F.** Description of parameters related to the human-mosquito coupling model.
(DOCX)
