# Supplementary material for: Spatiotemporal expansion of Aedes aegypti and the dengue fever epidemic under climate change in China
Source: PLoS Negl Trop Dis. 2025 Nov 19;19(11):e0013702. doi: 10.1371/journal.pntd.0013702 (PMC12629432; doi:10.1371/journal.pntd.0013702)
Supplement: S1 Appendix — (DOCX) [file pntd.0013702.s001.docx]

**S1 Appendix**

**Description of mosquito growth and dengue transmission.**

1. **The mosquito life cycle**

**Egg hatching stage**

The temperature threshold for this stage is set at 14.59°C^[1]^. If the daily average temperature on a given day is ≥ 14.59°C and the accumulated GDD since the eggs were laid reaches 42.4, the eggs will hatch. There is a positive correlation between temperature and hatching time; that is, at warmer temperatures, the hatching time is shorter.

**Immature development stage (larvae and pupae)**

After reaching the conditions for egg hatching, the larvae must accumulate a certain amount of GDD to complete the aquatic immature stage and emerge as adult mosquitoes. The temperature threshold is set at 11.78°C, and the GDD is set at 126.38.

**Blood feeding stage**

This stage refers to the time from the emergence of the adult mosquito to its first blood meal. During this period, *Aedes aegypti* is very sensitive to temperature. If the daily average temperature is between 16°C and 20°C, they will start searching for a host for a blood meal after 4 days; if the temperature is between 20°C and 26°C, they will take 2 days; if between 26°C and 35°C, they will take 1 day; and if the daily average temperature is > 35°C, they will take 2 days.

**Egg laying stage**

After completing the first blood meal, the adults undergo a temperature-sensitive gonotrophic cycle (GC) to gestate and lay eggs. If the daily average temperature on a given day is between 20°C and 26°C, they will start laying eggs after 8 days; if the temperature is between 26°C and 30°C, they will take 3 days; if between 30°C and 35°C, they will take 2 days; and if the daily average temperature exceeds 35°C, they will take 4 days.

**(2) Temperature effect**

In reality, *Aedes aegypti* may experience a wintering phase, and winter temperatures are a key factor affecting mosquito reproduction. Therefore, in the egg-laying stage of *Aedes aegypti*, the “cold kill” conditions have been considered: if, during the winter, the daily average temperature remains below a specified “cold kill” temperature threshold for a certain number of days, the eggs will die and thus be unable to continue their lifecycle. Based on studies by other researchers, the baseline model is set to <0°C for a duration of 152 days.

Additionally, this paper considers that the entire lifecycle may experience high-temperature phases in summer or autumn, hence a “heat-kill” condition has been established for all stages mentioned above. A high temperature threshold has been set: if the temperature exceeds this threshold, any life stage (including the aquatic stage and adult stage) will die directly, and development cannot proceed. Literature data indicate that the maximum temperature threshold conditions for egg hatching and larval development are ≥36°C and ≥36.5°C, respectively, while the maximum temperature threshold for the adult stage is >37°C. Therefore, this paper sets the “heat-kill” condition for any life stage of *Aedes aegypti* in the lifecycle to a daily temperature exceeding 38°C for 1 day.

In addition to considering temperature, this paper also incorporates precipitation as a constraint in the model, based on literature. The precipitation threshold is set at an annual rainfall of less than 200 mm to exclude areas that are too dry to support the survival of *Aedes aegypti*.

The daily egg production per female ($\phi$) peaks at temperatures between 25-30°C; the duration of the oviposition period ($l_{v}$) and the pupal conversion rate ($\sigma_{p}$) both peak at temperatures between 20-30°C; the larval conversion rate ($\sigma_{l}$) peaks at temperatures between 30-40°C. The mortality rate of larvae ($\mu_{l}$) and the mortality rate of adult mosquitoes ($d_{a}$) reach their lowest points at temperatures between 10-20°C; the mortality rate of pupae ($\mu_{p}$) is lowest at temperatures between 20-30°C, indicating that moderately high temperatures are most beneficial for the daily survival of *Aedes aegypti*.

1. **Dengue transmission process and related parameters**

Sum of these categories (S, E, I and R) represents the total population of the block, denoted as $\text{N}_{\text{h}}\text{=}\text{S}\text{+}\text{E}\text{+}\text{I}\text{+}\text{R}$, with the assumption that each $\text{N}_{\text{h}}$ is a constant. In the previous section, the life cycle of *Aedes aegypti* was divided into seven stages. Dengue transmission occurs only during the blood-feeding stage, where interactions between humans and the vector take place. To simulate dengue transmission, the adult stage is further divided into non-infected adult mosquitoes ($\text{A}_{\text{1}}$) and infected adult mosquitoes ($\text{O}_{\text{I}}$).

$\text{λ}_{\text{M}}$ is defined as a function of the biting rate ($\text{c}_{\text{v}}$), the proportion of the infected population (I), the probability of humans transmitting the virus to mosquitoes ($\text{β}_{\text{1}}$), and the susceptibility of the mosquitoes ($\text{σ}_{\text{M}}$). Therefore, $\text{λ}_{\text{M}}\text{=}\text{c}_{\text{v}}\text{σ}_{\text{M}}\text{β}_{\text{1}}\text{I}\text{/}\text{N}_{\text{h}}$. For humans, $\text{λ}_{\text{1}}$ is defined as a function of $\text{c}_{\text{v}}$, $\text{O}_{\text{I}}$, its infectivity ($\text{β}_{\text{M}}$), and human susceptibility ($\text{σ}_{\text{1}}$), $\text{λ}_{\text{1}}\text{=}\text{c}_{\text{v}}\text{σ}_{\text{1}}\text{β}_{\text{M}}\text{O}_{\text{I}}\text{/}\text{N}_{\text{h}}$.

**Related to egg production rate (**$\boldsymbol{\phi}$**，**$\boldsymbol{p}_{\boldsymbol{w}}$**)**

The number of eggs laid by *Aedes aegypti* is determined by several factors: the proportion of females laying eggs in moist habitats ($p_{w}$), the number of adult females ready to lay eggs ($A_{2}$), and the daily egg production per female ($\phi$). For the parameter $\phi$, it increases with rising temperatures but shows a declining trend once temperatures exceed the optimal range.

**Related to egg conversion rate (**$\boldsymbol{\sigma}_{\boldsymbol{ed}}$**，**$\boldsymbol{\sigma}_{\boldsymbol{ew}}$**)**

The likelihood of eggs being laid in moist or dry areas depends on the proportion of adult females laying eggs in moist habitats. While eggs can survive for months under dry conditions, they must be submerged in water to transition to the next stage. It is assumed that *Aedes aegypti* primarily breed in artificial containers, and human activities, such as lawn watering, reservoir accumulation, and providing water for animals, are the main sources of water needed for their aquatic breeding sites, causing dry eggs ($E_{d}$) to be converted into wet eggs ($E_{w}$), with this daily conversion rate denoted as $\sigma_{ed}$. For eggs laid in moist areas, as well as those that were dry but subsequently soaked, the conversion rate from the egg stage to the larval stage is denoted as $\sigma_{ew}$.

**Related to egg mortality rate (**$\boldsymbol{\mu}_{\boldsymbol{ed}}$**，**$\boldsymbol{\mu}_{\boldsymbol{e}\boldsymbol{w}}$**)**

Scholars Faull and Williams found that, on average, *Aedes aegypti* eggs can survive 187.4 days under dry conditions and 229.3 days under moist conditions. Consequently, the mortality rates for dry and wet eggs are calculated as 0.0053 ($\mu_{ed}$) and 0.0044, respectively. Considering that many cities in our country regularly clean standing water and conduct periodic mosquito and egg extermination activities, the mortality rate for wet eggs is set at 0.005 ($\mu_{ew}$).

**Related to the conversion rate of larvae and pupae (**$\boldsymbol{\sigma}_{\boldsymbol{l}}$**，**$\boldsymbol{\sigma}_{\boldsymbol{p}}$**)**

The conversion rate parameters for the larvae and pupae stages of *Aedes aegypti* are temperature-dependent. Tun-Lin et al. [2] studied the conversion time and survival rates at five temperatures between 15-35°C, finding that as the temperature increases, the conversion time decreases.

**Related to the mortality rate of larvae and pupae (**$\boldsymbol{\mu}_{\boldsymbol{l}}$**，**$\boldsymbol{\mu}_{\boldsymbol{p}}$**)**

Assuming a stable environment, larvae may compete for food and other resources, leading to density-dependent mortality, represented as $\frac{\mu_{l}L}{K}$, or by the inherent natural mortality rate $\mu_{l}$, where K is the environmental carrying capacity of the breeding site. The mortality rate for pupae is denoted as $\mu_{p}$.

**Related to the conversion rate and mortality rate of adults (**$\boldsymbol{n}_{\boldsymbol{a}}$**，**$\boldsymbol{d}_{\boldsymbol{a}}$**，**$\boldsymbol{l}_{\boldsymbol{v}}$**)**

In the adult stage, female *Aedes aegypti* need to feed on blood to provide nutrients for egg-laying. According to observations by Costa et al.[3], mosquitoes do not lay eggs during the first three days after becoming adults, so the conversion rate from the blood meal stage ($A_{1}$) is noted as $n_{a}$ = 1/3. The daily mortality rate ($d_{a}$) during the adult stage is temperature-dependent. The transition from the blood meal stage to the egg-laying stage ($A_{2}$) varies based on temperature, and Costa et al. fitted the egg-laying cycle data to a temperature-related Gaussian function ($l_{v}$). For $A_{2}$, whether starting another blood meal period or completing the final egg-laying period ($A_{3}$), the exit rate from the egg-laying stage is recorded as $\frac{1}{l_{v}}$.

The biting rate ($C_{v}$) of each female *Aedes aegypti* peaks in the temperature range of 30-35°C. The infectivity ($\beta_{1}$) when infected humans transmit the dengue virus to female *Aedes aegypti* peaks in the temperature range of 20-30°C. The infectivity ($\beta_{M}$) when infected female *Aedes aegypti* transmit the dengue virus to humans peaks in the temperature range of 25-35°C, indicating that moderately high temperatures are most favorable for the spread of dengue.

**References**

1. Iwamura T, Guzman-Holst A, Murray KA. Accelerating invasion potential of disease vector Aedes aegypti under climate change. Nat Commun. 2020 May 1;11(1):2130.
2. Tun-Lin W, Burkot TR, Kay BH. Effects of temperature and larval diet on development rates and survival of the dengue vector Aedes aegypti in north Queensland, Australia. Med Vet Entomol. 2000;14(1):31-7.
3. Pedrosa de Almeida Costa, E.A., et al., Impact of small variations in temperature and humidity on the reproductive activity and survival of Aedes aegypti (Diptera, Culicidae). Revista Brasileira de Entomologia, 2010. 54(3): 488-493.
