# Supplementary material for: Spatiotemporal expansion of Aedes aegypti and the dengue fever epidemic under climate change in China
Source: PLoS Negl Trop Dis. 2025 Nov 19;19(11):e0013702. doi: 10.1371/journal.pntd.0013702 (PMC12629432; doi:10.1371/journal.pntd.0013702)
Supplement: S2 Appendix — Fig A. Geographical distribution of the six case-study cities across China. Fig B. Flowchart of Aedes aegypti population model. Fig C. Dynamics flowchart of dengue virus transmission at the human - Aedes aegypti Interface. Fig D. Sensitivity of Aedes aegypti abundance and dengue fever cases to temperature variations under the SSP370 Scenario (Guangzhou, 2060). (DOCX) [file pntd.0013702.s002.docx]

**S2 Appendix**

**
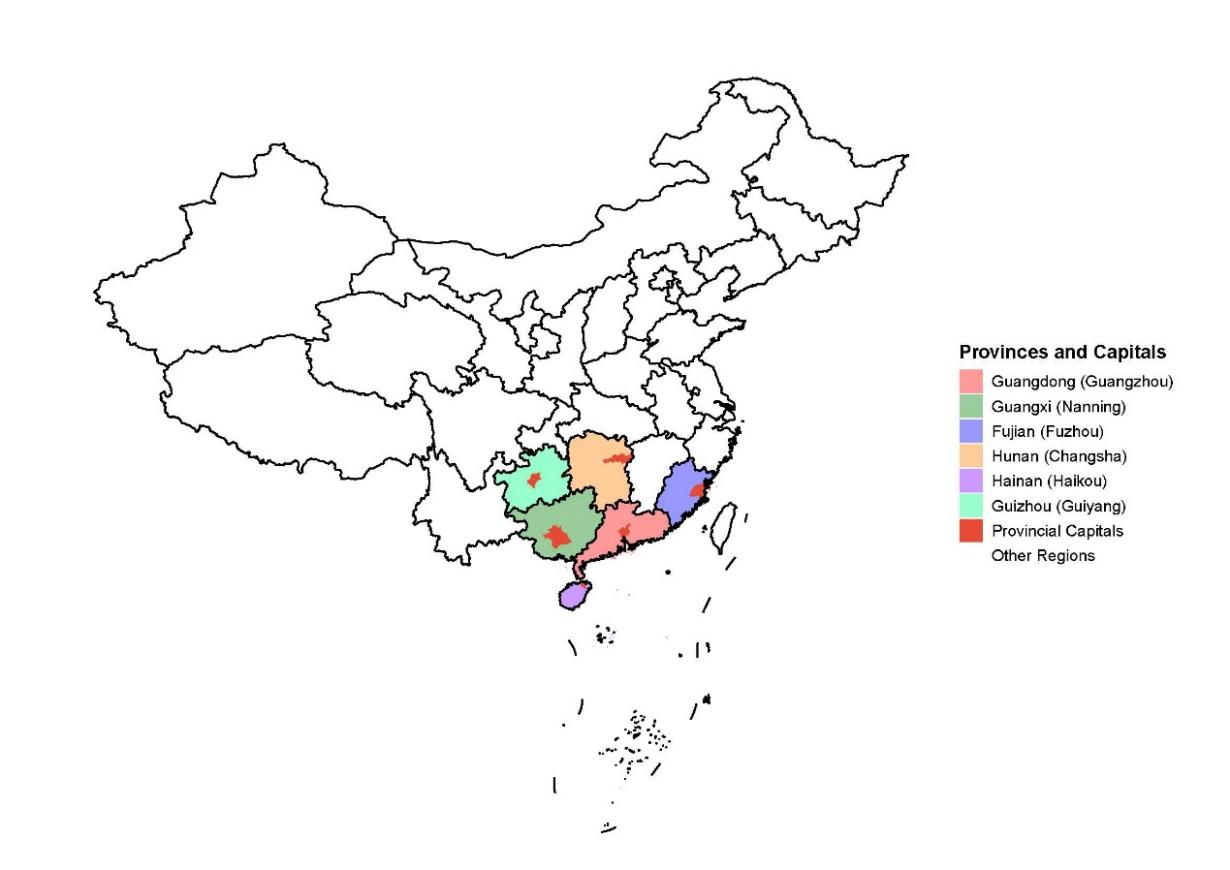
**

**Note:** The base map of China (including the national boundary and coastline) was obtained from the Standard Map Service of the Ministry of Natural Resources of the People’s Republic of China (http://bzdt.ch.mnr.gov.cn/) and is authorized for public use and publication. The locations of the six cities are marked on the map.

**Fig A.** Geographical distribution of the six case-study cities across China.


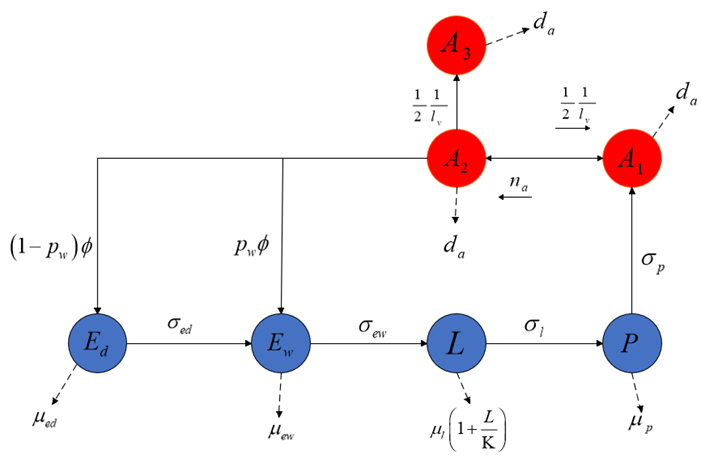


**Fig B.** Flowchart of *Aedes aegypti* population model. Life begins with the egg, which hatches into a larva in water at suitable temperatures. The larvae develop into pupae, and then emerge as adults. Adult females feed on human or animal blood to produce eggs. Population increases at each biological stage result from maturation from the previous stage, while decreases due to maturation to the next stage or mortality.


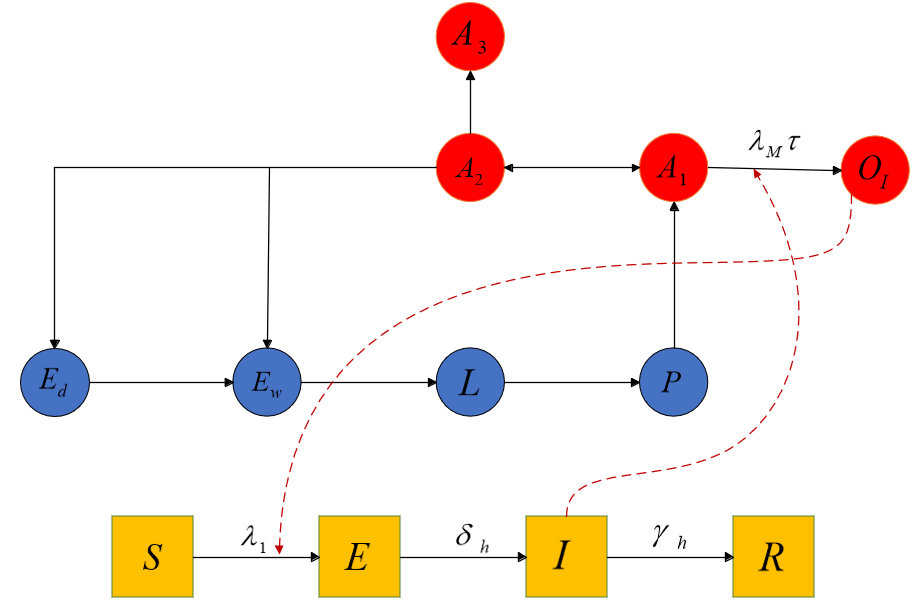


**Fig C.** Dynamics flowchart of dengue virus transmission at the human - *aedes aegypti* Interface.


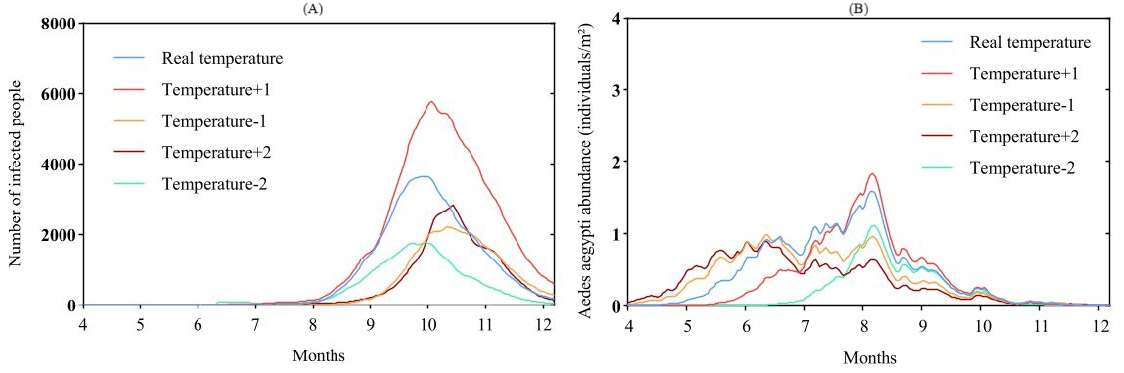


**Fig D.** Sensitivity of *Aedes aegypti* abundance and dengue fever cases to temperature variations under the SSP370 Scenario (Guangzhou, 2060). The blue line represents the abundance simulation under real temperature conditions, while the other lines represent simulations with daily temperature increases or decreases (±1°C or ±2°C) from April to December.
