# Supplementary material for: Spatiotemporal expansion of Aedes aegypti and the dengue fever epidemic under climate change in China
Source: PLoS Negl Trop Dis. 2025 Nov 19;19(11):e0013702. doi: 10.1371/journal.pntd.0013702 (PMC12629432; doi:10.1371/journal.pntd.0013702)
Supplement: S3 Appendix — Table A. Summary of shared socioeconomic pathways. Table B. Variable description in NEX-GDDP-CMIP6. Table C. Description of variables related to Aedes aegypti. Table D. Description of parameters related to Aedes aegypti. Table E. Description of variables related to the human-mosquito coupling model. Table F. Description of parameters related to the human-mosquito coupling model. (DOCX) [file pntd.0013702.s003.docx]

**S3 Appendix**

**Table A.** Summary of shared socioeconomic pathways

| Scenarios | Summary of SSP narratives | Radiative forcing |
| --- | --- | --- |
| **SSP126** (Sustainability - taking the green road (Low challenges to mitigation and adaptation)) | The global trajectory is gradually shifting towards a more sustainable direction, prioritizing inclusive development while respecting perceived environmental limits. This transition is driven by a growing commitment to achieving development objectives, leading to reduced inequality both among and within nations. Consumption patterns are evolving towards lower material growth and decreased resource and energy intensity. | 2.6 W/m^2^ of radiative forcing by 2100 |
| **SSP370** Regional rivalry - A rocky road (High challenges to mitigation and adaptation) | Over time, policies increasingly pivot towards national and regional security challenges. Countries prioritize attaining energy and food security targets within their regions, often at the expense of broader developmental initiatives. Investments in education and technological advancement decline, resulting in sluggish economic growth. Population growth rates vary, with industrialized nations experiencing low growth while developing countries see higher rates. Insufficient international attention to environmental issues contributes to severe environmental degradation in certain regions. | 7.0 W/m^2^ of radiative forcing by 2100 |
| **SSP585** Fossil-fueled development - Taking the highway (High challenges to mitigation, low challenges to adaptation) | Global markets are increasingly integrated. There are also strong investments in health, education, and institutions to enhance human and social capital. At the same time, the push for economic and social development is coupled with the exploitation of abundant fossil fuel resources and the adoption of resource and energy intensive lifestyles around the world. All these factors lead to rapid growth of the global economy, while global population peaks and declines in the 21st century. | 8.5 W/m^2^ of radiative forcing by 2100 |

**Table B.** Variable description in NEX-GDDP-CMIP6

| Variable | Description | Unit |
| --- | --- | --- |
| pr | Precipitation (including liquid and solid phases) | kg/m^2^/s |
| tmax | Maximum near-surface air temperature | K |
| tmin | Minimum near-surface air temperature | K |
| tm | Average near-surface air temperature | K |

**Table C.** Description of variables related to *Aedes aegypti*

| Symbol | Numerical attribute | Unit | Meaning |
| --- | --- | --- | --- |
| $E_{d}$ | Variable | Value | Dry egg population |
| $E_{w}$ | Variable | Value | Wet egg population |
| $L$ | Variable | Value | Larval population |
| $P$ | Variable | Value | Pupal population |
| $A_{1}$ | Variable | Value | Blood meal population |
| $A_{2}$ | Variable | Value | Egg-laying population |
| $A_{3}$ | Variable | Value | Post-oviposition population |
| $t$ | Independent Variable | day | Time (origin: January 1) |

**Table D.** Description of parameters related to *Aedes aegypti*

| Constant parameter | |  |  |  |
| --- | --- | --- | --- | --- |
| Symbol | Numerical value | Unit | Meaning | Origin |
| $\sigma_{ed}$ | 0.4 | day^-1^ | Conversion rate from dry eggs to wet eggs | - |
| $\sigma_{ew}$ | 0.596 | day^-1^ | Conversion rate from wet eggs to larvae | Focks et al. (1993)^[1]^ |
| $\mu_{ed}$ | 0.0053 | day^-1^ | Mortality rate of the dry egg population | Faull and Williams (2015)^[2]^ |
| $\mu_{ew}$ | 0.005 | day^-1^ | Mortality rate of the wet egg population | - |
| $n_{a}$ | 1/3 | day^-1^ | Conversion rate of the blood meal population | Costa et al. (2010)^[3]^ |
| $H_{t}$ | 10.5 | hours /day | Number of hours during the day | - |
| $L_{max}$ | 300 | mg m^-2^ | Maximum larval biomass | Tompkins AM et al. (2013)^[4]^ |
| Temperature-related parameters | |  |  |  |
| Symbol | Expression | | Meaning | Origin |
| $\phi$ | $78.86e^{-\left(\frac{T-29.21}{8.041}\right)^{2}}/7$ | | Number of eggs laid by each female per day | Costa et al. (2010)^[3]^ |
| $l_{v}$ | $4.727e^{-\left(\frac{T-26.16}{7.574}\right)^{2}}$ | | Duration of the oviposition period | Costa et al. (2010)^[3]^ |
| $e_{s}$ | $0.6108e^{\frac{17.27T}{T+237.3}}$ | | Saturated vapor pressure | Gbenga et al. (2016)^[5]^ |
| $\sigma_{l}$ | (1) | | Conversion rate from larvae to pupae | Jing et al. (2019)^[6]^ |
| $\sigma_{p}$ | (2) | | Eclosion rate of pupae | H. M. YANG et al. (2009)^[7]^ |
| $\mu_{l}$ | (3) | | Mortality rate of larvae | Jing et al. (2019)^[6]^ |
| $\mu_{p}$ | (4) | | Mortality rate of pupae | Jing et al. (2019)^[6]^ |
| $d_{a}$ | (5) | | Mortality rate of adults | Jing et al. (2014)^[8]^ |

$\sigma_{l}=\left\{\begin{matrix} 9.0064\cdot10^{-5},T<11\text{℃} \\ \begin{matrix} -1.847+0.8291T-0.1457T^{2}+0.01304T^{3}-6.461\times10^{-4}T^{4} \\ +1.796\times10^{-5}T^{5}-2.617\times10^{-7}T^{6}+1.551\times10^{-9}T^{7} \end{matrix}\text{，}others \end{matrix}\right.$ (1)

$\sigma_{p}=\left\{\begin{matrix} 9.0064\cdot10^{-4},T<10\text{℃} \\ \text{0,}T>40\text{℃} \\ \begin{matrix} 0.131-0.05723T+0.01164T^{2}-1.341\times10^{-3}T^{3} \\ +8.723\times10^{-5}T^{4}-3.071\times10^{-6}T^{5}+5.153\times10^{-8}T^{6} \\ -3.423\times10^{-10}T^{7} \end{matrix}\text{,}others \end{matrix}\right.$ (2)

$\mu_{l}=\min\left(2.32-0.419T+0.0273T^{2}-7.53\times10^{-4}T^{3}+7.5\times10^{-6}T^{4},1\right)$ (3)

$\mu_{p}=\min\left(0.425-0.0325T+7.06\times10^{-4}T^{2}+4.39\times10^{-7}T^{3},1\right)$ (4)

$d_{a}=\min\left(0.869-0.159T+0.0112T^{2}-3.41\times10^{-4}T^{3}+3.81\times10^{-6}T^{4},1\right)$ (5)

**Table E.** Description of variables related to the human-mosquito coupling model

| Symbol | Numerical Attribute | Unit | Meaning |
| --- | --- | --- | --- |
| $O_{I}$ | Variable | Value | Infected *Aedes aegypti* population |
| $S$ | Variable | Value | Susceptible population |
| $E$ | Variable | Value | Latent population |
| $I$ | Variable | Value | Infected population |
| $R$ | Variable | Value | Recovered population |

**Table F.** Description of parameters related to the human-mosquito coupling model

| Constant Parameter | |  |  |  |
| --- | --- | --- | --- | --- |
| Symbol | Numerical Value | Unit | Meaning | Origin |
| $\tau$ | 0.1 | day^-1^ | Extrinsic incubation period | Watts et al. (1987)^[9]^ |
| $\sigma_{M}$ | 1 | - | Susceptibility of *Aedes aegypti* | Paula et al. (2011)^[10]^ |
| $\sigma_{1}$ | 1 | - | Susceptibility of humans | Paula et al. (2011)^[10]^ |
| $\delta_{h}$ | 1/5 | day^-1^ | Intrinsic incubation period | Wearing et al. (2006)^[11]^ |
| $\gamma_{h}$ | 1/7 | day^-1^ | Infection recovery rate | Nishiura et al. (2006)^[12]^ |
| Temperature-related Parameters | |  |  |  |
| Symbol | Expression | | Meaning | Origin |
| $c_{v}$ | (1) | | Biting rate | Max et al. (2023)^[13]^ |
| $\beta_{1}$ | (2) | | Infectivity | Max et al. (2023)^[13]^ |
| $\beta_{M}$ | (3) | | Infectivity | Max et al. (2023)^[13]^ |

$c_{v}=\left\{\begin{matrix} 0.000202T\left(T-13.35\right)\sqrt{40.08-T},13.35\text{℃}\leq T\leq40.08\text{℃} \\ 0,\text{ }others \end{matrix}\right.$ (1)

$\beta_{1}=\left\{\begin{matrix} 0.001044T\left(T-12.286\right)\sqrt{32.461-T},12.286\text{℃}\leq T\leq32.461\text{℃} \\ 0,\text{ }others \end{matrix}\right.$ (2)

$\beta_{M}=\left\{\begin{matrix} 0.0729T-0.9037,12.4\text{℃}\leq T\leq26.1\text{℃} \\ 1,26.1\text{℃}<T\leq32.5\text{℃} \\ 0,\text{ }others \end{matrix}\right.$ (3)

**References**

1. Focks DA, Haile DG, Daniels E, Mount GA. Dynamic life table model for Aedes aegypti (Diptera: Culicidae): analysis of the literature and model development. J Med Entomol. 1993 Nov;30(6):1003-17. pmid: 8271242.
2. Faull KJ, Williams CR. Intraspecific variation in desiccation survival time of Aedes aegypti (L.) mosquito eggs of Australian origin. J Vector Ecol. 2015 Dec;40(2):292-300. pmid: 26611964.
3. Pedrosa de Almeida Costa, E.A., et al., Impact of small variations in temperature and humidity on the reproductive activity and survival of *Aedes aegypti* (Diptera, Culicidae). Revista Brasileira de Entomologia, 2010. **54**(3): 488-493.
4. Tompkins AM, Ermert V. A regional-scale, high resolution dynamical malaria model that accounts for population density, climate and surface hydrology. Malar J. 2013 Feb 18;12:65. pmid: 23419192.
5. Abiodun GJ, Maharaj R, Witbooi P, Okosun KO. Modelling the influence of temperature and rainfall on the population dynamics of Anopheles arabiensis. Malar J. 2016 Jul 15;15:364. pmid: 27421769.
6. Liu-Helmersson J, Brännström Å, Sewe MO, Semenza JC, Rocklöv J. Estimating Past, Present, and Future Trends in the Global Distribution and Abundance of the Arbovirus Vector *Aedes aegypti* Under Climate Change Scenarios. Front Public Health. 2019 Jun 12;7:148. pmid: 31249824.
7. Yang HM, Macoris ML, Galvani KC, Andrighetti MT, Wanderley DM. Assessing the effects of temperature on the population of Aedes aegypti, the vector of dengue. Epidemiol Infect. 2009 Aug;137(8):1188-202. pmid: 19192322.
8. Liu-Helmersson J, Stenlund H, Wilder-Smith A, Rocklöv J. Vectorial capacity of Aedes aegypti: effects of temperature and implications for global dengue epidemic potential. PLoS One. 2014 Mar 6;9(3):e89783. pmid: 24603439.
9. Watts DM, Burke DS, Harrison BA, Whitmire RE, Nisalak A. Effect of temperature on the vector efficiency of Aedes aegypti for dengue 2 virus. Am J Trop Med Hyg. 1987 Jan;36(1):143-52. pmid: 3812879.
10. Luz PM, Lima-Camara TN, Bruno RV, Castro MG, Sorgine MH, Lourenço-de-Oliveira R, et al. Potential impact of a presumed increase in the biting activity of dengue-virus-infected Aedes aegypti (Diptera: Culicidae) females on virus transmission dynamics. Mem Inst Oswaldo Cruz. 2011 Sep;106(6):755-8. pmid: 22012232.
11. Wearing HJ, Rohani P. Ecological and immunological determinants of dengue epidemics. Proc Natl Acad Sci U S A. 2006 Aug 1;103(31):11802-7. pmid: 16868086.
12. Nishiura, H., Mathematical and statistical analyses of the spread of Dengue*.* Dengue Bulletin. 2006 Dec; 30: 51-67. https://iris.who.int/handle/10665/170261.
13. Ramírez-Soto MC, Machuca JVB, Stalder DH, Champin D, Mártinez-Fernández MG, Schaerer CE. SIR-SI model with a Gaussian transmission rate: Understanding the dynamics of dengue outbreaks in Lima, Peru. PLoS One. 2023 Apr 13;18(4):e0284263. pmid: 37053225.
